# Randomised comparison of a balloon-expandable and self-expandable valve with quantitative assessment of aortic regurgitation using magnetic resonance imaging

**DOI:** 10.1007/s12471-020-01414-0

**Published:** 2020-04-03

**Authors:** N. H. M. Kooistra, M. Abawi, M. Voskuil, K. Urgel, M. Samim, F. Nijhoff, H. M. Nathoe, P. A. F. M. Doevendans, S. A. J. Chamuleau, G. E. H. Leenders, T. Leiner, A. C. Abrahams, H. B. van der Worp, P. Agostoni, P. R. Stella

**Affiliations:** 1 Department of Cardiology, University Medical Centre Utrecht, Utrecht, The Netherlands; 2 Department of Cardiology, St. Antonius Hospital, Nieuwegein, The Netherlands; 3 Netherlands Heart Institute (ICIN), Utrecht, The Netherlands; 4 Department of Radiology, University Medical Centre Utrecht, Utrecht, The Netherlands; 5 Department of Nephrology and Hypertension, University Medical Centre Utrecht, Utrecht, The Netherlands; 6 Department of Neurology and Neurosurgery, Brain Centre Rudolf Magnus, University Medical Centre Utrecht, Utrecht, The Netherlands

**Keywords:** Aortic valve stenosis, Balloon-expandable heart valve, Self-expandable heart valve, Transcatheter aortic valve implantation, Quantitative aortic regurgitation, Magnetic resonance imaging

## Abstract

**Introduction:**

Transcatheter aortic valve implantation (TAVI) is a safe and effective treatment for inoperable, intermediate- or high-risk patients with severe symptomatic aortic stenosis and has been associated with excellent clinical outcomes. A clinically relevant remaining problem is aortic regurgitation (AR) post-TAVI, which is associated with increased mortality. Therefore, we conducted a prospective randomised trial to assess the safety and efficacy of a first-generation self-expandable valve (SEV; CoreValve) and a third-generation balloon-expandable valve (BEV; Sapien 3) with respect to clinical outcomes and AR as determined quantitatively by magnetic resonance imaging (MRI).

**Methods:**

The ELECT study was an investigator-initiated, single-centre trial involving patients with severe symptomatic aortic stenosis and with a clinical indication for transfemoral TAVI. Fifty-six patients were randomly assigned to the BEV or SEV group.

**Results:**

AR determined quantitatively by MRI was lower in the BEV than in the SEV group [regurgitant fraction: 1.1% (0–8.0) vs 8.7% (3.0–14.8) for SEV; *p* = 0.01]. Secondary endpoints according to the criteria of the Second Valve Academic Research Consortium (VARC-2) showed BEV to have better early safety [0 (0%) vs 8 (30%); *p* = 0.002] at 30 days and a lower risk of stroke [0 (0%) vs 5 (21%); *p* = 0.01], major adverse cardiac and cerebrovascular events [0 (0%) vs 10 (38%); *p* < 0.001] or death [0 (0%) vs 5 (19%); *p* = 0.02] in the 1st year compared with SEV.

**Conclusions:**

The use of the latest generation of BEV was associated with less AR as quantitatively assessed by MRI. Although the use of MRI to quantify AR is not feasible in daily clinical practice, it should be considered as a surrogate endpoint for clinical outcomes in comparative studies of valves for TAVI.

ClinicalTrials.gov number NCT01982032.

## What’s new?

- We report on a randomised trial comparing the use of the latest-generation balloon-expandable valve and a first-generation self-expandable valve in patients undergoing transcatheter aortic valve implantation.
- The severity of aortic regurgitation (AR) was quantitatively assessed by magnetic resonance imaging (MRI).
- The latest-generation balloon-expandable valve was associated with less AR compared with the first-generation self-expandable valve.
- Assessing AR quantitatively by MRI should be considered as a surrogate endpoint for clinical outcomes in comparative studies of valves for TAVI.

## Background

Transcatheter aortic valve implantation (TAVI) is a safe and effective treatment for inoperable, intermediate- or high-risk patients with severe symptomatic aortic stenosis and has been associated with excellent clinical outcomes [1, 2]. A remaining problem is post-TAVI aortic regurgitation (AR). Moderate or severe AR after TAVI is associated with an increased risk of mortality [3]. Therefore, reducing AR after TAVI remains an important target to optimise treatment. Wide ranges of AR rates have been reported, partly due to non-standardised and non-quantitative measurements [3–5], resulting in under-or overestimation of AR [6–8] as well as inter-observer variability. So far, quantitative phase-contrast magnetic resonance imaging (MRI) has proved to be the most accurate method for assessing AR and the best method to improve the understanding of the relation between AR severity and clinical outcomes [9, 10].

Over the past decade, several improvements to address AR have been introduced in different valves with different expansion systems. Two often-used systems are the self-expandable valve (SEV) by Medtronic (CoreValve, Medtronic, Minneapolis, MN, USA) and the balloon-expandable valve (BEV) by Edwards (Sapien 3, Edwards Lifesciences, Irvine, CA, USA). The latter has been specifically designed with the aim of reducing paravalvular AR by an additional outer skirt. Numerous studies have been published on the safety, efficacy and post-procedural AR of the SEV and the latest-generation BEV [11–13]. To our knowledge, however, no randomised comparison between the two types of valve is available that investigates the differences in quantitatively measured AR and clinical outcomes.

Therefore, we conducted a prospective randomised trial to assess the safety and efficacy of the latest-generation BEV and first-generation SEV with respect to clinical outcomes, and AR as determined by MRI.

## Methods

The study design and patient selection of the Edwards Sapien 3 periprosthetic leakage evaluation versus Medtronic CoreValve in transfemoral aortic valve implantation (ELECT) trial have been described previously [14]. Briefly, this study was an investigator-initiated, single-centre, prospective, two-arm randomised controlled trial comparing the latest-generation BEV (Sapien 3) and the first-generation SEV (CoreValve), with the intention of including 106 patients according to block randomisation. The CoreValve was chosen for comparison, because of its widespread use at the time the trial was initiated. The study was approved by the local ethics committee, in accordance with the ethical principles of the Declaration of Helsinki, and written informed consent was obtained from all patients.

Primary endpoint was severity of post-procedural AR, quantitatively assessed by MRI, performed 4–5 days after the index procedure. The severity of AR with respect to regurgitant fraction was graded according to previously published definitions: none/trace <8%, mild 9–20%, moderate 21–39% and severe >40% [15]. As these definitions were based on patients without a TAVI valve, the flow values used for the regurgitant fraction were measured at the level of the left ventricular outflow tract for the aortic outflow volume, and at the level of the aorta ascending for the regurgitant volume. The aim was to obtain the most reliable measurements for patients post-TAVI according to Kooistra et al. [16], whose study reported accurate quantification of AR by MRI velocity mapping in the presence of a prosthetic valve, when corrected for a systematic error and when the appropriate MRI slice position is used. Their findings were applied to the present study. For the regurgitant volume, a linear regression correction was applied [for Sapien 3: (measured flow −3.3823)/0.9634; for CoreValve: (measured flow −0.3903)/1.0392], to achieve most accurate AR results based on ex vivo phantom experiments [16]. The regurgitant fraction was calculated by aortic outflow volume/corrected aortic regurgitant volume × 100% offline using PACS (Sectra PACS, version 17.3, Sectra Workstations IDS7, Linköping, Sweden).

Secondary clinical endpoints at 30 days and 1 year have been described previously and were defined as per the Second Valve Academic Research Consortium (VARC-2) definitions [14, 17]. All patients were actively followed up for 1 year, and had clinical visits and transthoracic echocardiographic (TTE) evaluations at 6 months and 1 year. Quality of life was assessed on average 1 month before and 1 year after the index procedure by the Short-Form-36 and EuroQol visual analogue scale, and new cerebral lesions were measured by cerebral MRI (performed at day 4–5 after the index procedure).

All endpoints were analysed on an intention-to-treat basis. Continuous variables were presented as means ± standard deviation or medians (interquartile range) and compared using the two-sided unpaired Student *t*-test or Mann-Whitney U test, depending on data distribution. Categorical variables were presented as counts and percentages and compared using Fisher’s exact test. Time-to-event curves were constructed using Kaplan-Meier curves and compared with the log-rank test. The paired *t*-test, repeated-measures analysis of variance and Wilcoxon signed rank test were used to assess the change in effective regurgitant orifice area and mean aortic gradient over time between and within groups. The correlations between continuous and ordinal variables were assessed using Spearman’s correlation coefficient. Sample size calculation has been described previously [14]. In summary, the calculation yielded a sample size of 49 patients in each arm to detect a difference between BEV over SEV with respect to AR with a power of 80%.

All tests were two-sided, and a *p*-value of less than 0.05 was regarded to be statistically significant. Data were analysed using IBM SPSS Statistics software version 21 (IBM Corp., Armonk, NY, USA).

## Results

### Study cohort

**Fig. 1:**
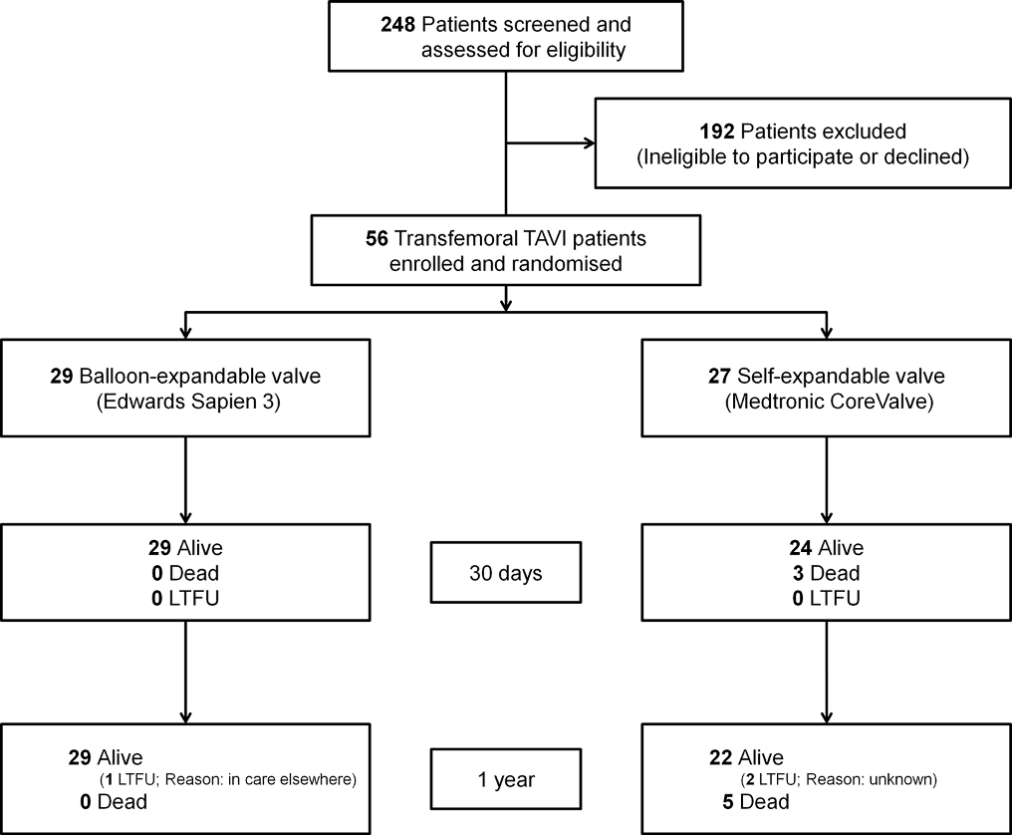
Study flowchart. *LTFU* lost to follow up, *TAVI* transcatheter aortic valve implantation

In total, 56 patients were included and randomly assigned to receive either SEV (*n* = 27) or BEV (*n* = 29) between January 2014 and May 2016 (Fig. 1). Study enrolment was preliminary halted, due to the EU release of a new-generation SEV (Medtronic Evolut) and slow enrolment.

The groups were well balanced in terms of baseline characteristics (Tab. 1). All patients received the assigned TAVI device and were followed-up for 1 year (Fig. 1). In total, 13 patients were excluded from MRI analysis (7 post-procedural pacemaker implantation, 2 pre-procedural pacemaker, 1 flow measuring error, 2 mortality and 1 clinically unstable), and in 8 out of 43 patients MRI measurements were performed only at the level of the aorta ascending and therefore reported without a correction factor.

**Table 1:** Baseline characteristics

	Number (%) of patients^a^		
	Balloon-expandable valve (*n* = 29)	Self-expandable valve (*n* = 27)	*p*-value
*Demographics*			
Age, years	77 ± 11	81 ± 7	0.32
Gender, female	15 (52)	13 (48)	1.00
BMI, kg/m^2^	28 ± 5	27 ± 5	0.31
Logistic EuroSCORE	13 ± 8	16 ± 10	0.43
NYHA III–IV	16 (55)	13 (48)	0.79
Coronary artery disease	17 (59)	10 (37)	0.12
Prior myocardial infarction	7 (24)	7 (26)	1.00
Prior CABG	5 (17)	3 (11)	0.71
Prior PCI	10 (35)	9 (33)	1.00
Prior stroke	5 (17)	4 (15)	1.00
Estimated GFR, ml/min	65 ± 20	61 ± 23	0.54
Diabetes mellitus	6 (21)	11 (41)	0.15
Hypertension	23 (79)	15 (56)	0.09
Dyslipidaemia	18 (62)	18 (67)	0.79
Peripheral artery disease	3 (10)	6 (22)	0.29
Atrial fibrillation	10 (34)	7 (26)	0.57
Chronic pulmonary disease	1 (3)	7 (26)	0.02
Permanent pacemaker	2 (7)	0	0.49
QOL, EQ-VAS, mean (95% CI)	57 (48.8–65.7)	57 (49.6–65.2)	0.98
*Transthoracic echocardiography*			
Aortic valve area, cm^2^	0.72 ± 0.2	0.75 ± 0.3	0.90
Mean gradient, mm Hg	42 ± 13	44 ± 20	0.80
Left ventricle ejection fraction, %	53 ± 10	51 ± 11	0.54
Regurgitation, moderate or severe			
– Aortic	4 (14)	0	0.11
– Mitral	3 (10)	6 (23)	0.28

^a^Plus-minus values are mean ± SD

*BMI* body mass index, *NYHA* New York Heart Association, *CABG* coronary artery bypass grafting, *PCI* percutaneous coronary intervention, *GFR* glomerular filtration rate, *QOL* quality of life, *IQR* interquartile range, *EQ-VAS* EuroQol-visual analogue scale, *CI* confidence interval

### Quantitative aortic regurgitation

**Fig. 2:**
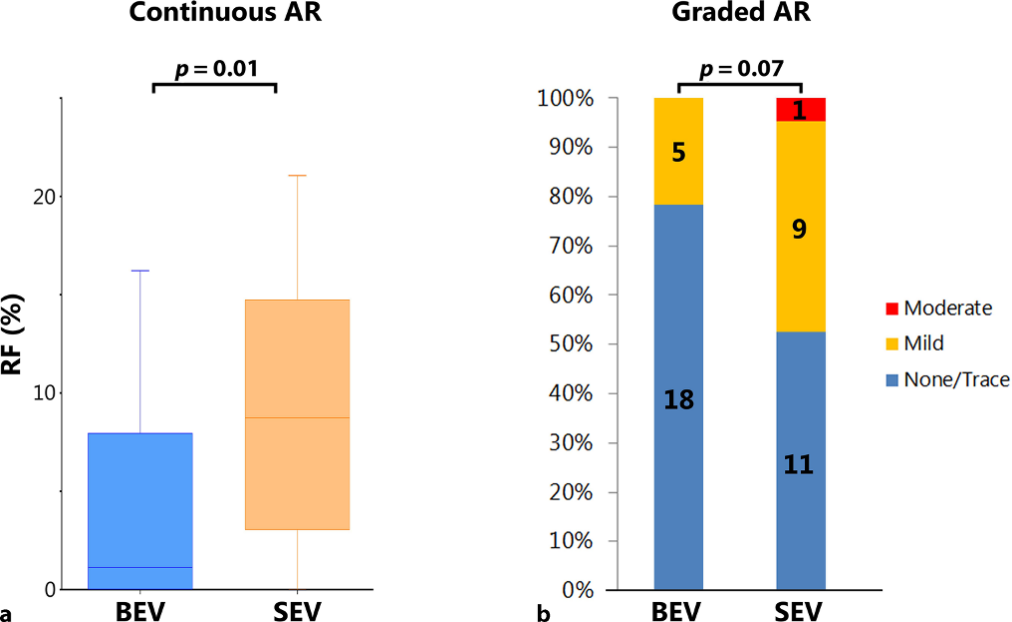
Quantitative assessment of aortic regurgitation (*AR*) with magnetic resonance imaging compared per valve type (BEV, SEV). **a** Boxplot of the continuous quantitative result. **b** Quantitative results stratified into AR grades. *RF* regurgitant fraction, *BEV* balloon-expandable valve, *SEV* self-expandable valve

The AR quantitatively measured by MRI was assessed in 43 patients (BEV: *n* = 23; SEV: *n* = 20) and showed a significant difference in favour of BEV for the regurgitant fraction [1.1% (0–8.0) vs 8.7% (3.0–14.8) for SEV; *p* = 0.01; Fig. 2]. After stratification into AR grades, this difference was not significant (*p* = 0.07; Fig. 2). With MRI we measured only the quantitative amount of regurgitation and not the origin; therefore, no distinction between valvular and paravalvular AR could be made for this modality.

The semi-quantitatively assessed severity of AR on TTE was still significantly lower in patients treated with the BEV at 1‑year follow-up (0% moderate, 14% mild, 86% none/trace for BEV vs 6% moderate, 38% mild, 56% none/trace for SEV; *p* = 0.04; Tab. 3). All AR appeared to be paravalvular on TTE.

### Clinical outcomes at up to 1 year

Procedural characteristics are shown in Tab. 2. No significant intra-procedural differences were observed between the two groups, besides a shorter fluoroscopy time in the BEV group (18 ± 6 min vs 22 ± 9 min; *p* = 0.02).

**Table 2:** Procedural features

	Number (%) of patients^a^		
	Balloon-expandable valve (*n* = 29)	Self-expandable valve (*n* = 27)	*p*-value
General anaesthesia	1 (3)	0	1.00
Pre-dilatation	29 (100)	26 (96)	0.48
Valve size, mm			
– 23	7 (24)	0	<0.001
– 26	13 (45)	4 (15)	
– 29	9 (31)	18 (67)	
– 31	0	5 (19)	
Post-dilatation	7 (24)	12 (44)	0.16
Valve malpositioning	0	1 (4)	0.48
Implantation of ≥2 valves	0	1 (4)	0.48
Fluoroscopic time, min	18 ± 6	22 ± 9	0.02
Radiation, mGy	521 ± 279	664 ± 324	0.07
Contrast volume, ml	163 ± 42	165 ± 40	0.72
Intraprocedural mortality	0	2 (8)	0.22
Device success (VARC-2)	27 (93)	21 (78)	0.14

^a^Plus-minus values are mean ± SD

*VARC‑2* Valve Academic Research Consortium‑2

At 30 days, there was a trend towards lower stroke rates among BEV patients compared with SEV patients [0 (0%) vs 3 (12%); *p* = 0.06; Tab. 3]. At 1 year, the difference in stroke rate between the groups had increased by 9% [BEV: 0 (0%) vs SEV: 5 (21%); *p* = 0.01; Tab. 3].

**Table 3:** Clinical outcome at 30 days and 1 year with balloon-expandable or self-expandable valve (intention-to-treat population)

	30 days				1 year						
	Balloon-expandable valve (*n* = 29) *N* (%)	Self-expandable valve (*n* = 27) *N* (%)		*p-*value	Balloon-expandable valve (*n* = 29) *N* (%)		Self-expandable valve (*n* = 27) *N* (%)		*p*-value		
Death	0	3 (11)		0.07	0		5 (19)		0.02		
– Cardiovascular death	0	3 (11)		0.07	0		5 (19)		0.02		
Repeat hospitalisation	1 (3)	2 (8)		0.44	2 (7)		3 (13)		0.44		
– Valve re-intervention	0	0		–	0		0				
Stroke or TIA											
– All	3 (10)	3 (12)		0.79	3 (10)		6 (25)		0.19		
– Stroke	0	3 (12)		0.06	0		5 (21)		0.01		
a. Ischaemic	0	3 (12)		0.06	0		5 (21)		0.01		
b. Bleeding	0	0			0		0				
– TIA	3 (10)	0		0.09	3 (10)		3 (15)		0.82		
AMI	0	0			0		0				
Vascular complication											
– Major	0	2 (7)		0.14	0		3 (12)		0.06		
– Minor	1 (3)	1 (4)		0.96	1 (3)		1 (4)		0.96		
Bleeding											
– Life-threatening	0	2 (7)		0.14	0		2 (7)		0.14		
– Major	1 (3)	1 (4)		0.96	2 (7)		1 (4)		0.64		
– Minor	1 (3)	2 (7)		0.49	2 (7)		2 (7)		0.86		
Pacemaker implantation	5 (19)	6 (24)		0.61	5 (19)		7 (29)		0.40		
MACCE	0	7 (26)		0.003	0		10 (38)		<0.001		
Early safety at 30 days	0	8 (30)		0.002							
In-hospital stay, days	5 (5–6)	6 (5–7)		0.04							
AKI in hospital	2 (7)	2 (8)		1.00							
– Stage 1	2 (7)	2 (8)		1.00							
– >Stage 1	0	0									
Cerebral MRI lesions post-procedure	19/23 (83)	19/21(91)		0.67							
– Number per patient (IQR)	3(1–6)	4 (2.5–9)		0.16							
– Volume per patient mm^3^ (IQR)	235(37–701)	566(132–948)		0.08							
NYHA class improvement at 1 year					19 (68)		7 (35)		0.02		
Aortic regurgitation on TTE									0.04		
– None/trace					24 (86)		9 (56)				
– Mild					4 (14)		6 (38)				
– Moderate					0		1 (6)				
– Severe					0		0				
QOL, EQ-VAS											
– Score, mean (95% CI)					71 (64.2–77.1)		68 (60.0–75.4)		0.54		
Score change compared to baseline, mean (95% CI)					12 (3.3–21.2)		7 (−2.2–15.7)		0.39		

*TIA* transient ischaemic attack, *AMI* acute myocardial infarction, *MACCE* major adverse cardiac and cerebrovascular event, *AKI* acute kidney injury, *MRI* magnetic resonance imaging, *IQR* interquartile range, *NYHA* New York Heart Association, *TTE* transthoracic echocardiography, *QOL* quality of life, *EQ-VAS* EuroQol visual analogue scale, *CI* confidence interval

Binary events were calculated using Kaplan-Meier methods, and corresponding *p*-values using log-rank tests

**Fig. 3:**
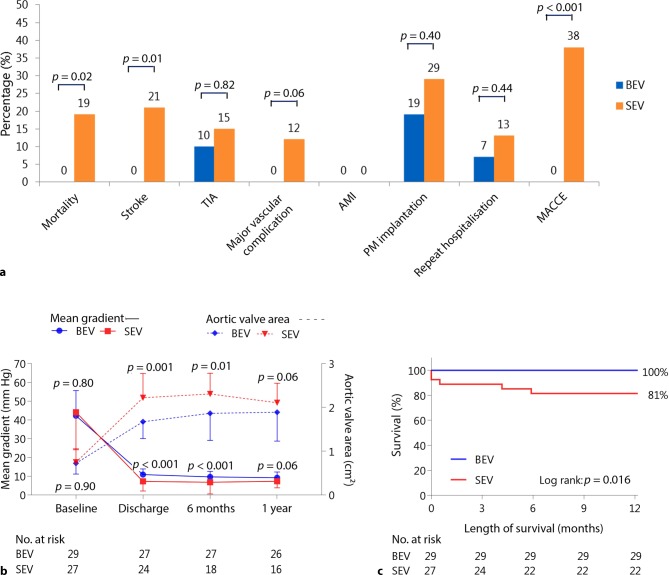
One-year clinical outcome. **a** One-year event rates of major VARC-2-related outcomes, comparison between valves. **b** Changes in aortic mean gradient and aortic valve area over four time points, with comparisons between the BEV and SEV group; between-group differences were significant at discharge and 6 months for both mean gradient and aortic valve area. *Error bars* represent 1 SD and are visualised one-sided. **c** Kaplan-Meier graph showing survival in BEV and SEV groups. *BEV* balloon-expandable valve*, SEV* self-expandable valve, *VARC‑2* Valve Academic Research Consortium‑2, *TIA* transient ischemic attack, *AMI* acute myocardial infarction, *PM* pacemaker, *MACCE* major adverse cardiac and cerebrovascular events

The composite endpoint 30-day early safety according to the VARC‑2 criteria was significantly better for the BEV group [0 (0%) vs 8 (30%); *p* = 0.002], as well as MACCE at 30 days and length of hospital stay (Tab. 3; Fig. 3a). At 1 year, MACCE rate was still significant lower for the BEV group [0 (0%) vs 10 (38%); *p* < 0.001]. Furthermore, all-cause mortality was significantly lower in the BEV group at 1 year [0 (0%) vs 5 (19%); *p* = 0.02; Fig. 3a, c]. All deaths were due to cardiovascular causes: one peri-procedural tamponade, two cases of terminal heart failure, one type A aortic dissection in combination with a spondylodiscitis and a high suspicion of septic endocarditis, and one stroke within the 1 year of follow-up.

At 1 year, clinical New York Heart Association (NYHA) symptoms had improved more in BEV patients compared with the SEV group (Tab. 3). Aortic mean gradient differed at baseline, but was comparable between the groups at 1 year (Fig. 3b).

No significant correlation between NYHA class improvement and AR was found (Spearman’s correlation coefficient 0.3; *p* = 0.09).

## Discussion

The ELECT trial is the first randomised controlled trial that compares quantitative AR as measured by MRI and clinical outcomes between the latest-generation balloon-expandable valve and a widely used first-generation self-expandable valve.

In this study, patients with a BEV had lower AR rates post-TAVI. Furthermore, clinical outcomes at 1 year showed a lower mortality rate, fewer strokes and MACCE, and a shorter hospital stay for the BEV group.

A distinguishing feature of the investigated latest-generation BEV to reduce post-TAVI AR is the additional skirt around the valve. Indeed, a lower AR rate for the new BEV was observed compared with the SEV. In recent studies of the latest-generation BEV, similar AR rates to those in our study have been reported (none/trace 56–74% [11, 12, 18]; moderate/severe 0–3.5% [11, 12, 18]). These AR rates are lower than those of the previous BEV generation (none/trace 29–66% [19–21]; moderate/severe 5–23% [3, 19–22]). The very low AR rates for BEV observed in the present study might reflect the effectiveness of the new skirt of this valve. Other potential factors influencing the reduced AR and complications might be the increased operator experience and the use of multi-detector computed tomography for adequate valve sizing.

The major strength of the present study is the fact that the AR severity was quantitatively assessed by MRI. A problem of previous studies is the wide range of AR rates, which are partly due to non-standardised and non-quantitative AR measurements that remain imprecise due to under- or overestimation of AR as well as inter-observer variability [4, 6–8]. Since MRI is currently considered to be the most accurate method available with low intra- and inter-observer variability and high accuracy, resulting in good reproducibility [9, 10, 16, 23, 24], the assessment and comparison of AR severity between the two valves has been assessed in this study as accurately as possible. The accuracy of AR is important, since AR is a frequent remaining problem that is associated with an increased 30-day and 1‑year mortality [3].

To investigate the association between quantitatively measured AR severity and clinical outcomes, NYHA class improvement was used, since the mortality rate was too low to study a correlation. No significant association was found in our study between AR severity and NYHA class improvement. This might be due to the low AR rates, with mostly none/trace or mild AR severity. However, this lack of association between mild AR and clinical outcomes is in accordance with the FRANCE‑2 Registry and the PARTNER II SAPIEN 3 trial [25, 26].

The secondary clinical endpoints of our randomised trial showed a significantly lower mortality rate at 1 year with no mortality at all in the BEV group. The mortality rate of 19% for the SEV group is in line with other studies of this first-generation valve [13, 19, 22, 27, 28]. The low mortality rate associated with the BEV might be explained by the reduction in complications that are associated with increased mortality, i.e. reduction in AR severity, strokes and life-threatening bleeding.

The striking low rates of stroke and life-threatening bleeding in the BEV group might be caused by many patient- or procedure-related factors. However, these could also be related to the low delivery profile (14 or 16 Fr), the lower crossing profile of the Sapien 3 system and a better distal flexion of the delivery catheter, resulting in fewer aortic arch injuries and life-threatening bleedings in patients receiving this latest-generation BEV. The overall low complication and mortality rates of the BEV are also reflected in the composite endpoints according to the VARC‑2 criteria, with a high device success rate, better early safety and a lower rate of MACCE. The observed difference in clinical outcomes between the SEV and BEV groups in our study might represent the difference in valve generation. Studies of the newer generations of the SEV have found lower complication rates, which are in line with the latest-generation BEV with a 1-year all-cause mortality rate and stroke rate around 8.5% and 3.5% respectively[29, 30].

### Study limitations

The results of the clinical outcomes in the present study should be interpreted in the context of its small sample size and the lack of power to detect differences in endpoints—due to low recruitment—between the valves, resulting in wider confidence intervals and possible underestimation of differences. Nevertheless, the reported differences that were statistically significant can be considered to be a real difference and not a coincidence, since the statistical type I error (false-positive statistically significant result; α = 0.05) is not related to the power of a test. However, as stated by Button et al. [31], it is possible that the magnitude of the found effect is exaggerated due to the underpowered endpoint, which might explain the high stroke rate in the SEV group. Secondly, in this study, the first-generation CoreValve was used, which is nowadays not employed anymore. However, at the time the trial was initiated, these were the two most common valves used commercially.

### Clinical implications and future perspectives

The results of this study show a high-quality comparison between two valve types regarding AR severity as quantitatively assessed by MRI. Although the use of MRI for the quantification of AR is not feasible in daily clinical practice due to its high costs, low availability and certain contra-indications (e.g. in patients with claustrophobia or < 6 weeks after implantation of pacemaker leads), it is of additive value as regards improving the accuracy and reproducibility of AR as well as reducing variability with respect to AR severity. In addition, studies with quantitative AR assessment are needed in order to improve the accuracy of AR estimation and to better understand the relationship between AR severity and clinical outcomes as well as differences between next-generation valves. Therefore, measuring AR by MRI should be considered in future studies comparing valves. Finally, this small study could be a preamble to a new randomised comparison between current-generation TAVI valves, but probably requires a larger sample size because of smaller differences in technical and clinical outcomes.

## Conclusions

The use of latest-generation BEV was associated with less AR as quantitatively assessed by MRI. Although the use of MRI to quantify AR is not feasible in daily clinical practice, it should be considered as a surrogate endpoint for clinical outcomes in comparative studies of valves for TAVI.

## Appendix

**Table 4:** Short Form (SF)-36 quality of life questionnaire results, with effect size^a^ per valve type

	Baseline			1‑Year				
	Balloon-expandable valve	Self-expandable valve		Balloon-expandable valve		Self-expandable valve		
	Mean baseline ±SD (%)	Mean baseline ±SD (%)	*p*-value	Mean Change	Effect Size	Mean Change	Effect Size	*p*-value
General health	52.9 ± 19	46.7 ± 17	0.23	5.0 ± 24	0.27	5.3 ± 20	0.31	0.97
Physical function	35.8 ± 28	36.1 ± 28	0.97	13.2 ± 25	0.47	6.8 ± 19	0.24	0.37
Vitality	51 ± 20	47.1 ± 23	0.54	7.2 ± 24	0.36	5.9 ± 20	0.26	0.85
Physical role	23 ± 33	23.3 ± 37	0.98	16.7 ± 46	0.50	15.8 ± 50	0.43	0.95
Mental health	66.6 ± 21	70 ± 16	0.54	6.2 ± 20	0.30	4.4 ± 16	0.28	0.74
Bodily pain	71 ± 31	74.5 ± 25	0.64	10.9 ± 27	0.36	−3.2 ± 20	−0.13	0.07
Social function	59.7 ± 32	60.3 ± 29	0.95	9.7 ± 34	0.31	10.6 ± 29	0.36	0.94
Emotional role	47.6 ± 48	62 ± 47	0.28	13.6 ± 53	0.28	7.3 ± 49	0.15	0.70

^a^ Effect sizes were calculated as the baseline score minus the 1‑year score, divided by the SD (standard deviation) of the baseline score

*p*-values represent the between valve comparisons
